# Laparoscopic total extraperitoneal (TEP) mesh repair for femoral hernia in a patient of child B liver cirrhosis with ascites. A case report

**DOI:** 10.1016/j.ijscr.2020.05.091

**Published:** 2020-06-12

**Authors:** Aditya Kumar, Manjunath Maruti Pol

**Affiliations:** Department of Surgical Disciplines, All India Institute of Medical Sciences, New Delhi, India

**Keywords:** TEP, Totally extra-peritoneal, Child B cirrhosis, Femoral hernia, Laparoscopic TEP

## Abstract

**Introduction:**

Femoral hernia is infrequently encountered in surgical practice and is even rare in patients with liver cirrhosis. Recurrent pain adds to the existing morbidity and affects the quality of life of these patients. Management of such cases had rather high rates of morbidity and mortality owing to hepatic decompensation. However, more recent studies have shown a significant improvement of the quality of life and improved rates of morbidity in cirrhotic patients with inguinal hernias post repair. These studies all included open hernia repair with preperitoneal approach with improved results in terms of morbidity and lower rates of recurrence postoperatively. However, when compared to laparoscopic repairs these have more postoperative complications, complication related re-operations, pain and recurrence rates. Keeping these in mind, the laparoscopic approach was considered in our patient which has not been described yet in literature for femoral hernia. The report is in line with the SCARE criteria. (Agha et al. (2018) [1]) The case report is registered with research registry (UID researchregistry5467).

**Presentation of case:**

A 40 year old female patient with Child B cirrhosis of liver with ascites was operated for a symptomatic left uncomplicated femoral hernia using standard three port laparoscopic total extraperitoneal repair with prolene mesh. She was discharged on postoperative day 2. She developed ascitic leak from the hypogastric port site in the late postoperative period which was managed conservatively. The patient has remained stable without recurrence at one year follow up.

**Conclusion:**

Laparoscopic TEP may be a safe option with no major adverse events in symptomatic femoral hernias. Further studies are needed to ascertain its role.

## Highlights

• Minimal access laparoscopic techniques may be performed safely in patients with femoral hernia with liver cirrhosis.
• Minimal access technique provide less postoperative pain, lower recurrence and early discharge.
• Extraperitoneal approaches are safe in hernia patients with ascites associated with liver failure.
• Improved anesthetic techniques and improved medications have improved outcomes of surgery in patients with liver cirrhosis.

## 1 Background

Femoral (or crural) hernias are less frequently encountered in surgical practice as compared to inguinal hernia and comprise 2–4% of all groin hernia repairs [2,3]. The incidence and natural history has not been documented clearly in cirrhotic patients with ascites [4]. However, poor quality of life and the risk of incarceration remains [5]. Traditionally surgical repair has been contraindicated in this set of patients owing to an increased risk of complications and hepatic decompensation leading to increased morbidity and mortality [6]. Recent studies however, have shown an improvement in the quality of life with low morbidity in cirrhotic patients undergoing inguinal hernia repair [7]. We describe a successful repair of a femoral hernia in a patient of Child B cirrhosis with ascites using the Laparoscopic Totally Extraperitoneal (TEP) method. The report is in line with the SCARE criteria. (Agha et al. (2018) [1]).

## 2 Case presentation

**Fig. 1:**
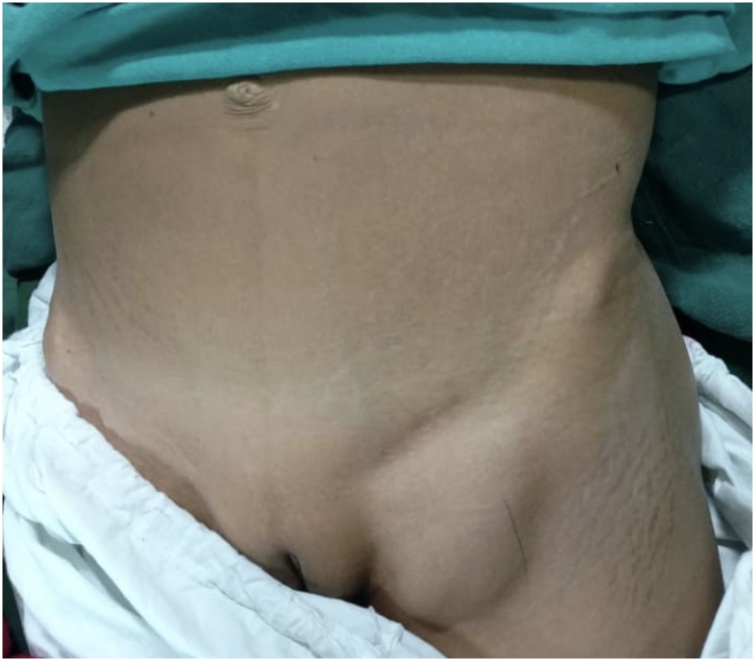
Left inguinal swelling.

**Fig. 2:**
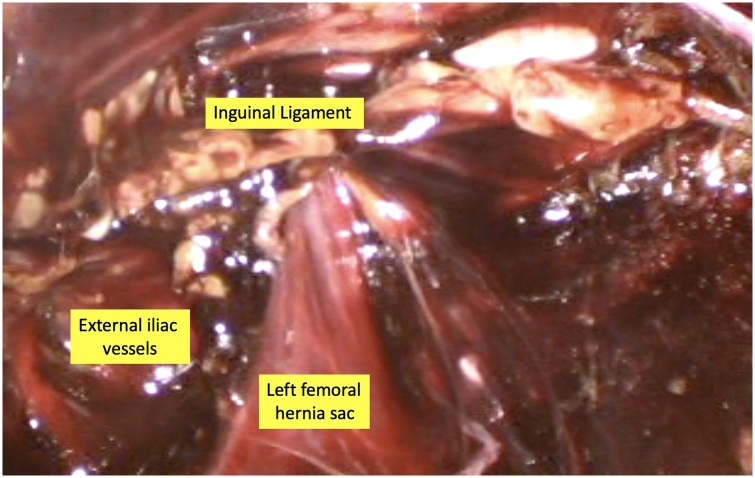
Sac seen herniating through femoral ring below the inguinal ligament.

A 40-year-old, female patient with Child B cirrhosis of liver with ascites was referred to us with a 4 × 3 cms left sided femoral hernia in the last three months (Fig. 1). The patient was symptomatic with intermittent episodes of pain, however, there was no evidence of incarceration. The patient was Child C at presentation and was stabilized to a Child B status with ascites by the gastroenterology team. She was then transferred to us, planned and taken up for a standard three port laparoscopic total extraperitoneal repair with a 15 × 15 cm prolene mesh under general anesthesia. We placed a 12 mm umblical port and two 5 mm ports under vision (one midway between the symphysis pubis and umbilicus and the other 2 in. above the symphysis pubis. We faced difficulty when we entered the preperitoneal space as ascitic fluid would push the peritoneum compromising space which eventually led to a small peritoneal hole (Fig. 2). We closed the hole using an endoloop and completed the procedure uneventfully. The perioperative stay were uneventful. She was discharged on the second post-operative day, but developed ascitic fluid leak from the hypogastric port site in the late postoperative period. It was successfully managed conservatively with dressings and improved by day 2. The patient has remained stable with no recurrence at one year follow up.

## 3 Discussion & conclusion

Femoral hernia is infrequently encountered in surgical practice and is even rare in patients with cirrhosis of liver [2]. There are also no accepted guidelines to manage such patients of femoral hernia with cirrhosis. Patients with symptoms such as recurrent pain add to the morbid process and affects the quality of life of patients in addition to the high risk of complications associated with femoral hernias [5]. Earlier reports on management of such cases were found to have rather high rates of morbidity and mortality [4,6]. However, more recent studies have shown a significant improvement of the quality of life and improved rates of morbidity in cirrhotic patients with inguinal hernias post elective repairs [7]. All previous studies have included open hernia repair as surgery of choice and the preperitoneal approach has been seen to have improved results in terms of morbidity and lower rates of recurrence postoperatively [8]. Open repairs have been performed more often with the belief that local anesthesia may be safer than using general anesthesia. However, these studies are old and more recent studies including improved anesthetic agents and use of minimally invasive techniques have shown improved outcomes with general anesthesia as well [9,10]. Also open Lichtenstein repairs have been known to have significantly increased rates of postoperative complications, complication related reoperations, pain and recurrence rates when compared to laparoscopic approach [3,11]. Keeping these findings in mind, and the fact that laparoscopic repair is routinely performed for most groin hernia at our centre, the laparoscopic approach was considered in our patient. Total Extraperitoneal Repair(TEP) was preferred over Transabdominal Preperitoneal repair(TAPP) to stay in the preperitoneal space which potentially prevents post-operative ascitic leak, bowel injury, postoperative adhesions and need for sutures in the peritoneal space [12]. Also TEP has less operation time as compared to TAPP at our center, preventing the potential anesthetic hazards [13]. While performing TEP, we faced two problems, one was increased oozing while dissecting planes and second was pressure created by ascitic fluid preventing adequate insufflation. Both these issues are related to the underlying disease process of CLD. Oozing led to depleted vision and associated decreased space due to ascites may have led to the breach in peritoneum. We were able to manage the breach with the endoloop suture and complete the surgery safely. The early perioperative course was uneventful and comfortable for the patient. There was some ascitic leak in the late postoperative period which may have been due to the peritoneal perforation intraoperatively. It was managed conservatively and took two days for control with conservative management. The laparoscopic extraperitoneal repair was performed successfully with no major complications providing sustained relief from symptoms and no recurrence. Similar findings have been noted in a recent article by Wang H et al. where they performed laparoscopic TEP for 17 patients with inguinal hernia and cirrhosis. They required no conversions, no perioperative mortality or recurrence of hernia at 24 months follow up. Four of their patients had intraoperative peritoneal breach which was managed conservatively and there were no major complications. [10] Hence, we conclude that laparoscopic TEP may be a safe option in the management of symptomatic femoral hernia and larger studies are needed to ascertain its role.

## Conflicts of interest

None.

## Sources of funding

None.

## Ethical approval

Case reports are exempt from ethical approval from Institutional review board.

## Consent

A copy of the written consent is available for review by the Editor-in-Chief of this journal on request.

## Research studies

1. Name of the registry: Research Registry
2. Unique identifying number or registration ID: researchregistry5467
3. Hyperlink to your specific registration (must be publicly accessible and will be checked): https://www.researchregistry.com/browse-the-registry#home/registrationdetails/5e83130dde185b0017853069/

## Guarantor

Dr. Manjunath Maruti Pol.

## Data & materials

Not applicable.

## Provenance and peer review

Not commissioned, externally peer-reviewed.

## CRediT authorship contribution statement

**Aditya Kumar:** Conceptualization, Data curation, Methodology, Resources, Writing - original draft, Writing - review & editing. **Manjunath Maruti Pol:** Conceptualization, Project administration, Writing - review & editing.

## References

[1] Agha R.A., Borrelli M.R., Farwana R., Koshy K., Fowler A.J., Orgill D.P. (2018). The SCARE 2018 statement: Updating consensus Surgical CAse REport (SCARE) guidelines. Int. J. Surg. Lond. Engl..

[2] Sucandy I., Kolff J.W. (2012). Incarcerated femoral hernia in men: incidence, diagnosis, and surgical management. North Am. J. Med. Sci..

[3] HerniaSurge Group (2018). International guidelines for groin hernia management. Hernia J. Hernias Abdom. Wall Surg..

[4] Belghiti J., Durand F. (1997). Abdominal wall hernias in the setting of cirrhosis. Semin. Liver Dis..

[5] Cox T.C., Huntington C.R., Blair L.J., Prasad T., Heniford B.T., Augenstein V.A. (2017). Quality of life and outcomes for femoral hernia repair: does laparoscopy have an advantage?. Hernia J. Hernias Abdom. Wall Surg..

[6] Carbonell A.M., Wolfe L.G., DeMaria E.J. (2005). Poor outcomes in cirrhosis-associated hernia repair: a nationwide cohort study of 32,033 patients. Hernia J. Hernias Abdom. Wall Surg..

[7] Patti R., Almasio P.L., Buscemi S., Famà F., Craxì A., Di Vita G. (2008). Inguinal hernioplasty improves the quality of life in patients with cirrhosis. Am. J. Surg..

[8] Elgohary H., Nawar Am, Zidan A., Abdelmofeed Am, Elwan Th, Abourizk Mi (2018). Surgical and functional outcome of pre-peritoneal repair of inguinal hernia in cirrhotic patient with mild to moderate ascites. Surg. Curr. Res..

[9] Abbas N., Makker J., Abbas H., Balar B. (2017). Perioperative Care of Patients With Liver Cirrhosis: A Review. Health Serv Insights [Internet]. https://www.ncbi.nlm.nih.gov/pmc/articles/PMC5398291/.

[10] Wang H., Fu J., Qi X., Sun J., Chen Y. (2019). Laparoscopic totally extraperitoneal (TEP) inguinal hernia repair in patients with liver cirrhosis accompanied by ascites. Bull. Sch. Med. Md.

[11] Köckerling F., Bittner R., Kofler M., Mayer F., Adolf D., Kuthe A. (2019). Lichtenstein versus total extraperitoneal patch plasty versus transabdominal patch plasty technique for primary unilateral inguinal hernia repair: a registry-based, propensity score-matched comparison of 57,906 patients. Ann. Surg..

[12] Vărcuş F., Duţă C., Dobrescu A., Lazăr F., Papurica M., Tarta C. (2016). Laparoscopic repair of inguinal hernia TEP versus TAPP. Chir. Buchar. Rom. 1990.

[13] Krishna A., Misra M.C., Bansal V.K., Kumar S., Rajeshwari S., Chabra A. (2012). Laparoscopic inguinal hernia repair: transabdominal preperitoneal (TAPP) versus totally extraperitoneal (TEP) approach: a prospective randomized controlled trial. Surg. Endosc..

